# Automated Structure Discovery for Scanning Tunneling Microscopy

**DOI:** 10.1021/acsnano.3c12654

**Published:** 2024-04-22

**Authors:** Lauri Kurki, Niko Oinonen, Adam S. Foster

**Affiliations:** † Department of Applied Physics, Aalto University, Aalto, Espoo 00076, Finland; ‡ Nanolayers Research Computing Ltd., London N12 0HL, U.K.; § WPI Nano Life Science Institute (WPI-NanoLSI), Kanazawa University, Kakuma-machi, Kanazawa 920-1192, Japan

**Keywords:** scanning probe microscopy, scanning tunneling microscopy, tip functionalization, machine learning, convolutional neural network, structure discovery

## Abstract

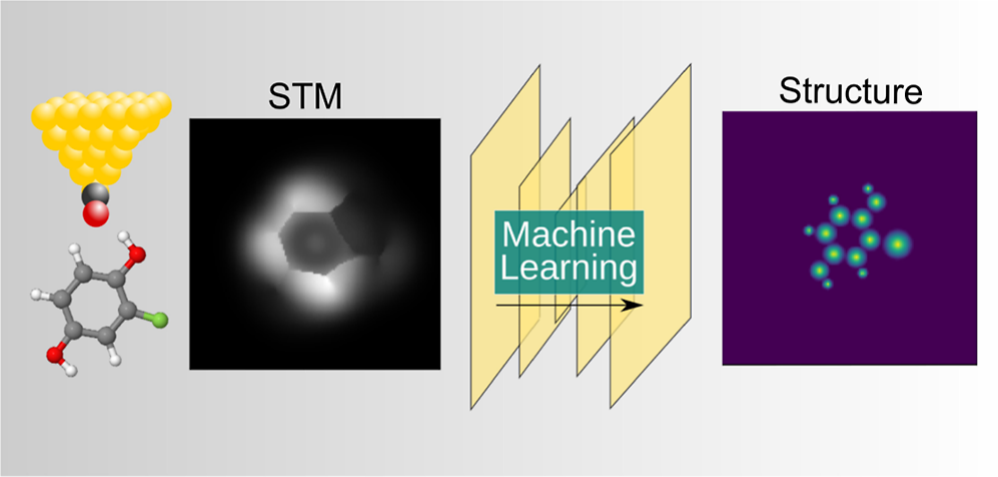

Scanning tunneling microscopy (STM) with a functionalized tip apex reveals the geometric and electronic structures of a sample within the same experiment. However, the complex nature of the signal makes images difficult to interpret and has so far limited most research to planar samples with a known chemical composition. Here, we present automated structure discovery for STM (ASD-STM), a machine learning tool for predicting the atomic structure directly from an STM image, by building upon successful methods for structure discovery in noncontact atomic force microscopy (nc-AFM). We apply the method on various organic molecules and achieve good accuracy on structure predictions and chemical identification on a qualitative level while highlighting future development requirements for ASD-STM. This method is directly applicable to experimental STM images of organic molecules, making structure discovery available for a wider scanning probe microscopy audience outside of nc-AFM. This work also allows more advanced machine learning methods to be developed for STM structure discovery.

Scanning probe microscopy (SPM) methods are powerful tools for studying nanoscale systems with atomic resolution. As the basis of SPM methods, scanning tunneling microscopy (STM)^1^ and atomic force microscopy (AFM)^2^ have been widely utilized in the characterization of various systems, such as biological samples, hybrid inorganic–organic interfaces, and individual steps of on-surface reactions.^3−9^ To enhance the spatial accuracy in the characterization, the probe tip can be functionalized using a chemically inert, flexible apex (often a CO molecule) to allow scanning at a very close tip-sample distance where Pauli repulsion is the dominating interaction.^10,11^ With respect to the detailed characterization of atomic structures, there exist many demonstrations of the improved spatial resolution of AFM scanning with functionalized tips,^9,12,13^ but STM is now also being increasingly used in this bond-resolved mode.^8,13−18^ STM in particular benefits significantly from tip functionalization as sharp submolecular features appear in the image, revealing both the molecular skeleton and the electronic structure in high detail within the same experiment—this is impossible using a bare metal tip^19^ or by high-resolution AFM. In addition to atomic structural characterization, STM with a functionalized tip offers interesting approaches for electronic structure characterization via Frontier orbitals of the tip apex^20^ by improving the visibility of molecular orbitals^21^ and by distinguishing nearby molecular states.^17^

In terms of structure characterization, the main limitations of STM are its inability to see beyond the closest atom to the tip and the insurmountable problem of chemical identification of the atoms. In fact, research using bond-resolved STM has so far been mostly limited to planar molecules consisting of only a priori known atomic species, and the recognition of an unknown sample can require an extensive search through all possible molecules and configurations. The same problems have been experienced in the field of noncontact AFM where recent advances in machine learning image analysis have been proven effective in structure discovery and chemical identification of single molecules and ice structures.^22−27^ Beyond machine learning, some of the challenges with respect to chemical identification can be overcome by combining tip-enhanced Raman spectroscopy with STM and AFM to achieve quantitative chemical sensitivity.^28^ For STM, advanced image analysis methods have so far not been developed for bond-resolved imaging but instead the focus has been on, e.g., defect detection,^29^ molecule keypoint detection,^30^ surface characterization,^31^ and atom manipulation^32^ as well as autonomous experiments.^33^ Machine learning methods have also been used for automating the tip conditioning^34^ and tip functionalization^35^ in STM. It is clear that the focus in general has been on larger-scale systems and processes, and there is a need for automated characterization methods for bond-resolved imaging with a CO tip.

In this work, we present automated structure discovery for STM (ASD-STM), a machine learning approach for structure characterization directly from experimentally bond-resolved STM images. With ASD-STM, we offer a solution to the problems in STM structure characterization while bridging the gap between image analysis methods for high-resolution AFM and STM. As STM with a functionalized tip is a more easily accessible characterization method compared to noncontact AFM,^36^ this work also makes ASD available for a wider SPM audience and allows more sophisticated methods to be developed for sample recognition in STM.

## Results

**Figure 1:**
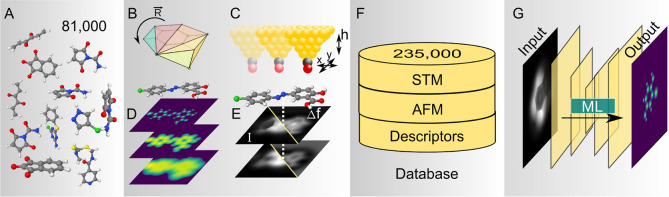
Scheme of ASD-STM. (A) Starting from a large set of organic molecules, (B–E) we simulate STM and AFM for different rotations of the molecules and calculate corresponding physical descriptors. (F) The images are stored in a database and (G) used as training material for a machine learning model for STM structure discovery.

The proposed method starting from a data set of molecule geometries and ending in a machine learning model that can predict atomic structure directly from experimental images is outlined in Figure 1.

### Predictions on Simulated Images

**Figure 2:**
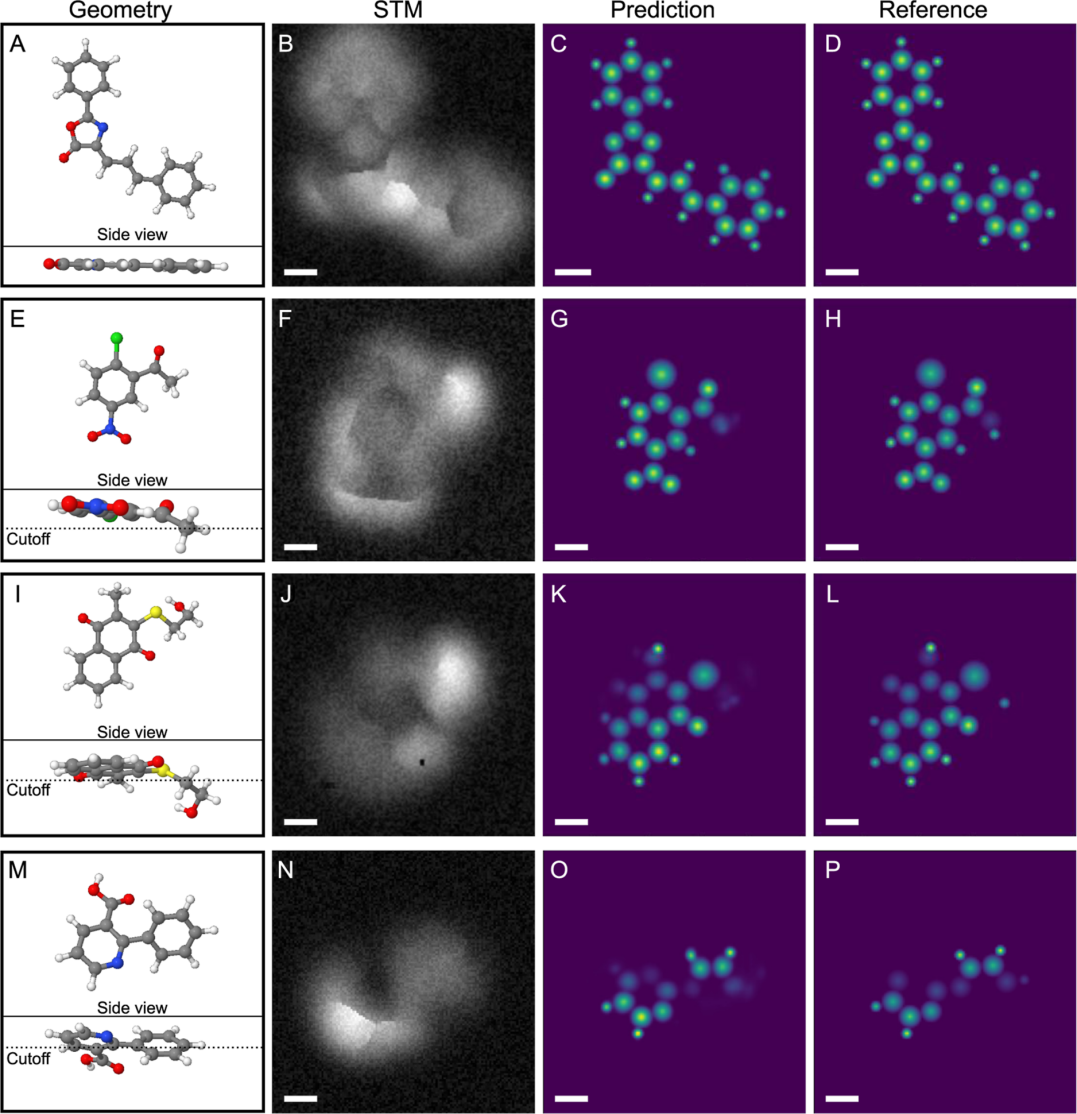
Example predictions of simulated STM images of (A–D) C_18_H_13_NO_2_, (E–H) C_8_H_6_ClNO_3_, (I–L) C_13_H_12_O_3_S, and (M–P) C_12_H_9_NO_2_S molecules from the testing set. Each molecule has a column for the geometry (descriptor depth cutoff shown in the side profile), STM image, predicted structure, and reference. Scale bar is 2 Å.

To benchmark our method and assess the improvement in prediction accuracy, we use the trained model to predict atomic structures from simulated images not included in the training data. The obvious benefit from analyzing simulated predictions is that the atomic coordinates of the reference, and therefore the image descriptor, are known exactly. Figure 2 shows three examples of predictions from simulated images. The three examples have been taken at varying scanning heights and the sample molecules are different in size and structure, and they contain different chemical species and functional groups to demonstrate the versatility of the method.

Molecule 1 (Figure 2A–D) is the largest of the three and it has two benzene groups and a 5-ring lactone group with an additional nitrogen heteroatom. The molecule is planar, and all atoms are visible to the tip resulting in the carbon backbone skeleton being revealed with the exception of the benzene rings which are not prominent in the STM image (Figure 2B). Even though the skeleton is not completely visible, the prediction (Figure 2C) is very close to that of the reference (Figure 2D). All atoms, including hydrogens which are not visible to the eye, are correctly located and the associated chemical identification distinguishes hydrogen atoms from carbon, nitrogen, and oxygen atoms. It is also promising that the model correctly identifies the oxygen bonded to the 5-ring as not hydrogen and that it does not blindly add hydrogen atoms to nitrogen and oxygen atoms of the 5-ring. Overall, the mean absolute error relative to the range of values in the reference is 1.1%.

The second example shows a smaller molecule containing a nitro group and a chlorine atom (Figure 2E–H). The molecule is slightly tilted, and it includes a nonplanar part in which the branch containing a carbonyl group is bending downward. This is projected in the reference descriptor by a barely visible carbon and two of the bonded hydrogen atoms are too deep to be considered (Figure 2H). Again, the prediction resembles the reference very closely (Figure 2G). The chlorine atom is distinguished from hydrogen atoms, and the relative height of the oxygen in the carbonyl group is correctly identified. The location of the rightmost carbon atom is predicted correctly in the scanning plane, but the disk is too bright, meaning that the vertical position prediction is wrong. Since the oxygen atom is approximately 1.0 Å higher than the deepest carbon atom, the disk representing it is very bright in comparison and it appears to shadow the nearby deep carbon atom, meaning that it is not surprising that the model struggles most in this region. The relative error is 0.4%.

The third simulated example contains a sulfur atom, a hydroxy group, and two carbon 6-rings, one of which has two carbonyl groups (Figure 2I–L). In the STM image, the sulfur atom appears as the brightest area (Figure 2J). This molecule is particularly tricky for chemical identification due to the carbonyl groups which could theoretically be hydrogen atoms of a benzene ring, and the sulfur atom bridging the two carbon structures could be, e.g., an oxygen atom forming an ether group. Regardless of the apparent challenges, the model predicts all of these properties correctly (Figure 2K). The disks representing oxygen atoms have a larger radius than the hydrogen atoms of the lower ring, and the sulfur atom has an even bigger radius distinguishing it from oxygen and carbon atoms. The carbon chain originating from the sulfur is not considered in the descriptor due to the depth threshold. Finally, all hydrogen atoms included in the reference are predicted and the tilting of the molecule is captured in the prediction. The relative error for this example is 0.6%. The fourth example consists of two 6-rings twisted with respect to each other, one containing a nitrogen heteroatom and a carboxyl group (Figure 2M–P). Here, the carbon backbone is predicted accurately despite the twisting, and the relative error is 0.7%. To illustrate the accuracy of three-dimensional (3D) molecules, we show more predictions in Supporting Information (see Figure S2). Overall, the prediction accuracy is very good on simulated STM images and the trained model predicts atomic structures of different sizes, distinguishes various chemical species, and even appears to predict seemingly hidden atoms. However, the final validation of the model has to be done on experimental data, and to this end, we first consider four benchmark molecules. Also, even though ASD-STM was trained on constant-height images, extending it to constant-current images could possibly enable easier 3D structure recognition. As a preliminary test of feasibility, we applied the present model to a constant-current image of benzene, and Figure S3 shows that the prediction is quite reasonable. Developing this further will certainly be a future target for the method.

### Experimental Validation

**Figure 3:**
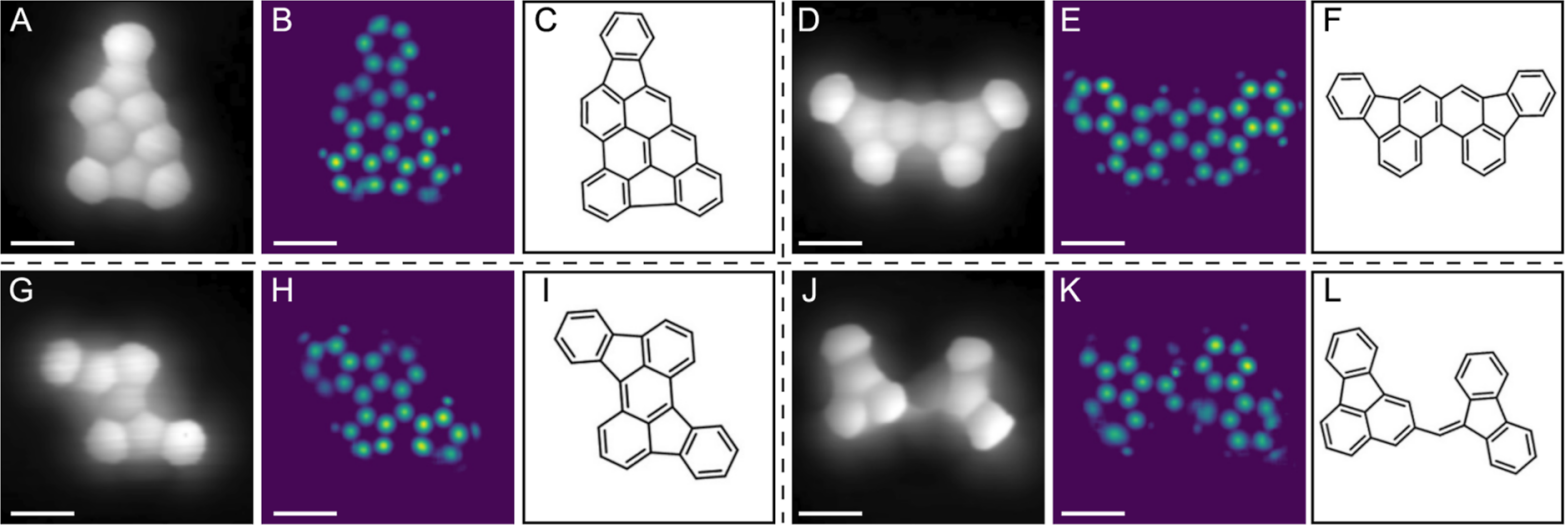
Predictions from four experimental STM images. (A,D,G,J) The first column of each example is the STM image used as input for ASD-STM. (B,E,H,K) The predictions are shown in the second column and (C,F,I,L) the third column contains the structural formula. Scale bar is 4 Å. STM images and structural formulas adapted with permission from ref (18) Ⓒ2020 ACS.

The first experimental images we apply ASD-STM on are STM images obtained by Song et al.^18^ that reveal the molecular skeletons of four hydrocarbons in high detail making them excellent for benchmarking purposes (Figure 3A,D,G,J). The molecules consist of exclusively carbon and hydrogen atoms constructing five- and six-membered rings, barring the fourth example in which a carbon atom links two ring structures (Figure 3L). In all STM images, the rings appear quite distorted, and in many cases, it is not immediately clear how many carbon atoms constitute the particular ring, and therefore along with general structure discovery, we consider our objective to be to distinguish these rings. The first example consists of nine carbon rings of which seven- are six-membered. Most of the rings, seven out of nine, are correctly identified in the prediction (Figure 3B), characterization of one ring is unclear, and characterization of one ring is misclassified. The model also seems to have learned how rings of different sizes connect with each other. Additionally, the prediction suggests that the bottom and top parts of the molecule are slightly closer to the tip than the central parts, but these predictions are difficult to validate.

The second example is an STM image of a symmetric molecule with eight rings (Figure 3D–F). The five rings are fairly large in the image, and the rings at all ends of the molecule appear brighter than in the center. The prediction is very good in terms of the carbon structure with all atoms predicted correctly (Figure 3E). In this case, even many hydrogen atoms are included in the prediction. The third example we consider is smaller than the previous molecules (Figure 3G–I), and again, the model performs well in predicting the atomic positions of carbon atoms. In this case, the bottom row of rings proposes a challenge for the model as the six rings in the corners are misclassified as five rings. A possible explanation for the wrong prediction is that the rings are almost circular and lack characteristic corners. This is supported by the first example which exhibited similar features in the STM image and where similar problems were encountered. It is also worth noting that the STM image contains considerable noise in the form of horizontal lines, but the model succeeds in the prediction regardless. We attribute this to a successful augmentation process during the training phase, where noise and cut-outs were applied to the initially pristine simulated images.

The final example is different from the others in that it includes two discrete ring structures that are connected by carbon atoms (Figure 3J–L). It is clear that the model struggles with the molecule and does not predict the correct structure in this case. The rightmost part is mostly correct, although the model does not seem to fully recognize the rings, as some of the predicted atoms are blurry and slightly deformed. The leftmost part of the molecule has more errors, and only the central five ring is correctly predicted (Figure 3K). A general problem with many machine learning models is that their explainability is poor and it is often difficult to explain the reasons behind a certain prediction.^37^ One possible reason would be the particular structure of the molecule; molecules containing two separate but connected carbon ring structures are rare in the data set, meaning that the model has not had proper experience and thus fails in the prediction. Also, it is vital to train the model to withstand noise and other artifacts present in experimental images, which is illustrated by comparing to predictions of these structures from pristine simulated images (see Figure S4). Overall, the predictions correspond well to the molecular structures, and in total, 106 out of 116 (91.4%) carbon atoms and 23 out of 31 (74.2%) carbon rings are predicted correctly.

**Figure 4:**
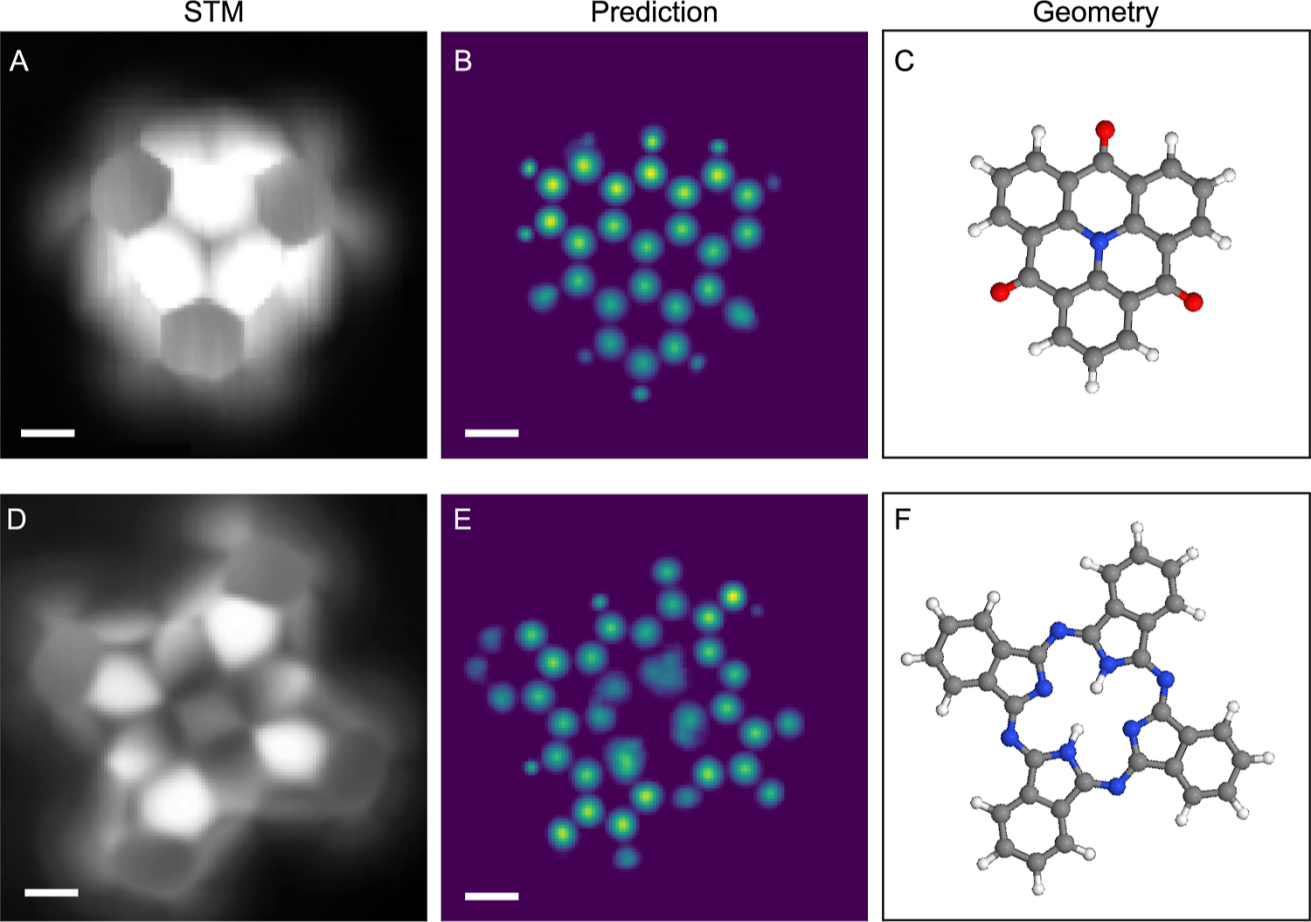
Prediction on the experimental image of the (A–C) TOAT (1,5,9-trioxo-13-azatriangulene) and (D–F) phthalocyanine (2*H*-Pc) molecules. For both molecules, the columns contain, from left to right, the STM image, structure prediction, and geometry of the molecule. Location of the pyrrolic hydrogen atoms in (F) as proposed in ref (21). Scale bar is 2 Å. STM images in (A) reprinted with permission from ref (38) Ⓒ2016 ACS and in (D) from ref (21) Ⓒ2023 ACS.

Predictions on the hydrocarbon examples are promising, but the molecular structures were rather simple, and we want to test the limits of the model on a more challenging example. To this end, we use STM images of the TOAT molecule (1,5,9-trioxo-13-azatriangulene) (Figure 4A)^38^ and phthalocyanine (2*H*-Pc) (Figure 4D).^21^ Predicting the atomic structure of TOAT is particularly interesting since the simulation software we used to generate the training data is known for not being able to properly reproduce experimental STM images of TOAT.^39^ Regardless, the prediction is in very good agreement with the true molecular structure (Figure 4B). The triangulene backbone is correctly identified, and all oxygen atoms are distinguished from hydrogen atoms, indicated by larger disks in the prediction. In this example, even most hydrogen atoms are located with the only exception being the top right ring missing one hydrogen. The disk representing the center nitrogen seems to have the same radius as the neighboring carbon atoms suggesting an incorrect prediction, but this classification is inconclusive. To assist in the chemical identification, we performed further analysis and trained another model which separates the disks into different classes by atomic type, which allowed us to distinguish the nitrogen heteroatom (Figure S5).^22^ Definitive chemical identification has been explored previously for AFM,^23,24^ but for STM, it remains a future challenge.

The most challenging example we test here is an STM image of 2*H*-Pc (Figure 4D), and this prediction (Figure 4E) reveals the limitations of our approach. 2*H*-Pc is a cyclic molecule consisting of four isoindole units connected by nitrogen atoms (Figure 4F). In this example, a perfect prediction would reveal the configuration of the hydrogen atoms in the central moiety. In the STM image, the inner five rings are bright and clearly exhibit the pentagon-like motif, whereas the outer six rings are less pronounced. The nitrogen atoms connecting the units appear as cones decaying toward the center. In general, the molecular skeleton is not very prominent in the image, and the signal originating from the electronic structure intersects significantly with the mechanical bending of the tip apex. It is apparent that these factors severely affect the ability of ASD-STM to make accurate predictions of the structure. In the prediction, the five rings apart from the top left ring are correctly identified, and all nitrogen atoms connecting the rings are located. The biggest errors are the missing halves of the six rings and also the central hydrogens that are not included in the prediction. It is worth noting that a similar phenomenon was observed in the case of the fourth hydrocarbon (Figure 3K) where the top half of a ring was missing in the prediction.

## Discussion

While predictions on most experimental examples were good, it is clear that there is room for improvement. Since the quality of data included in the training process is vital to the accuracy of predictions, rare structures affecting the accuracy should be discussed, as it raises the important question of how to select the molecules in the data set. In the scope of SPM structure discovery, there are two main approaches to data set generation. The first option, which this work highlights, is to create a large, diverse, and descriptive molecular data set of various chemical species and structures and to train a versatile model with the aim of predicting the structure of almost any small organic molecule. This approach has also been chosen in most previous sample characterization efforts (e.g.,^22,40^), but recently, another method has been used in ice structure discovery,^26,41^ where instead of a diverse data set, a tailored data set is utilized and perfected to make very accurate predictions possible in a constrained problem domain. That is, if the goal was to predict only the geometries of different hydrocarbons or triangulene-based molecules, the model would benefit from a tailored data set with a heavy emphasis on such structures. As methods for quick generation of molecular structures to create vast molecular data sets are becoming more readily available,^42−44^ the balance and cost-effectiveness of tailored versus diverse data sets should be the focus of future research.

Second, ASD-STM has been trained on synthetic STM images of molecules in isolation without the substrate due to much cheaper computational cost, and as such, the generated image data set should not be inferred as a source for accurate electronic structures of adsorbed organic molecules. Moreover, as we know that PPSTM cannot accurately reproduce STM images of the TOAT molecule, it was expected that ASD-STM would struggle with the experimental STM image of TOAT. However, the accuracy in the prediction was excellent, which suggests that even though images in the training set are not entirely representative of true images in terms of electronic structure, they capture the characteristic sharp lines coming from CO tip bending accurately, and this seems to be critical for structure discovery. Still, we do note that for systems experiencing significant charge transfer or orbital hybridization ASD-STM is most likely not a sufficient tool, and a more comprehensive approach is needed.^41^ Also, synthetic AFM images of isolated molecules in general correspond well to experimental AFM images where the substrate is naturally present, and since for some samples it is possible to simultaneously gather STM and AFM signals, the possibility of incorporating AFM data into the training process should be explored.

Third, we note that the appearance of the molecule in STM is sensitive to Frontier orbitals of the CO tip and consequently to the tip height as at closer tip-sample distances the tip exhibits a strong p-wave character which diminishes to a more s-wave character at longer distances.^21^ The s-wave character is also increased at higher absolute bias voltages.^45^ In this work, ASD-STM has been trained using a constant ratio of s and p-wave contributions, but the different orbital ratios should be accounted for if ASD-STM is used for a larger range of tip-sample distances or bias voltages. Finally, although it is inevitable that the accuracy of predictions on experimental images is worse than on simulated images, we will investigate this further in the future with a particular focus on improving the robustness of the model against experimental noise and artifacts.

## Conclusions

This work presents ASD-STM, a method for predicting the atomic structure of a sample molecule directly from a bond-resolved STM image. We present the workflow and the software required to generate an STM image data set and to train the ML model. Images included in the training set were synthesized by considering the molecules in isolation, and even though the electronic structure can be affected by the substrate, we showed that the approximation of molecules in isolation is reasonable for structure discovery on bond-resolved STM. The model was validated by applying it to experimental images of six different organic molecules, and on most samples, the accuracy was good in terms of atomic structure; additionally, we achieved a qualitative level of chemical identification by distinguishing between hydrogen, carbon, and oxygen atoms. While the chemical domain of the model is quite large within the class of organic molecules, many types of molecules such as organometallic compounds are not within the scope and would require expanding the training set. On the other hand, the example of 2*H*-Pc clearly demonstrated some of the challenges. The main limitation of the method is the reliance on the sharp submolecular features in the images, which restricts the method to high-quality images and to a range of short tip-sample distances. While the simulated images are augmented with noise and cut-outs during training, we anticipate that these augmentations are not the only difference between simulated and experimental images, and advanced augmentation strategies should be explored to make the model more robust against experimental conditions. Also, we discussed possible further improvements to ASD-STM by varying the orbital contributions in the tunneling calculation, by tailoring the composition of the initial data set of molecules to the problem at hand, and by including simultaneously gathered AFM data in the training process.

Despite the challenges, ASD-STM is readily applicable to STM images of various small organic molecules, and with this work, we allow more sophisticated methods to be developed for STM structure discovery. Finally, as bond-resolved STM images exhibit similar submolecular features as noncontact AFM images at a significantly reduced acquisition time, ASD-STM demonstrates a promising start for accelerating molecular structure discovery in SPM in general.

## Methods

### Simulated STM Images

The initial data set contains the optimized geometries and atomic point charges of approximately 81,000 small organic molecules with chemical species lighter than bromine (*Z* ≤ 35) with most emphasis on hydrogen, carbon, nitrogen, and oxygen atoms (Figure 1A).^25^ Simulating STM images requires that we first obtain the densities of states of all molecules in the data set, and since the molecular structures are already optimized, a single-point calculation is performed for all molecules. All ab initio calculations are performed using the FHI-aims code^46,47^ with the PBE functional^48^ considering only the Γ *k*-point. The unit cell is constructed by padding the molecule with 7 Å of empty space on each side to ensure that the periodic images do not interact with and affect the electronic structure.

The STM simulations for all molecules are done using the PPSTM code.^39^ PPSTM is based on the Bardeen tunneling theory,^49^ and it simulates constant height raster scanning by calculating the tunneling current at each point of a 3D grid of tip positions allowing for a quick simulation of multiple constant height scans at different tip-sample distances (Figure 1C,E). In PPSTM, the tunneling current is composed of contributions from the individual states of the tip and the sample. The sample states are obtained from density functional theory calculations as mentioned, and they are represented by Lorentzian distributions with a 0.1 eV broadening. The tip orbitals are considered as s and p orbitals located at the tip apex, and they provide independent tunneling channels. The tunneling channels are given a weight between 0 and 1, and the correct weights for a certain tip apex are found by matching with experiments (e.g., approximately 10% s and 90% p orbitals for CO tip^8,50^). Parallel to the STM simulations, we run the PPAFM^51,52^ code to calculate the relaxation of the CO molecule near the sample, and the relaxed positions are, in turn, used in the STM simulation to achieve a good resemblance to experimental images revealing the molecular skeleton. PPAFM calculates the CO relaxation by considering the CO molecule as a probe particle (PP) and using a mechanical model taking into account the Lennard-Jones and electrostatic potentials between the PP and the sample. The position of the PP approaching the sample is relaxed by minimizing the total force acting upon the PP, and while calculating the relaxed tip positions, we simultaneously obtain AFM images of the molecules with little additional cost by integrating the forces affecting the PP over its path.^53^ The AFM images are included in the data set of synthetic images, and they could be used as additional signal in a method developed for simultaneous AFM/STM imaging.

To maximize the amount of information in the SPM images, we emphasized flat regions normal to the scanning direction and also ensured a more even distribution of elements in the resulting images. To this end, we subjected the molecules to a set of rotations calculated separately for each molecule (Figure 1B). The rotations were obtained by computing the convex hull of the atomic coordinates, which defines planar segments of the molecule, and the rotations were weighted to include an even distribution of elements within 0.7 Å of the plane. After applying the rotations, our data set consists of synthetic SPM images of approximately 48,000 unique molecules and 235,000 unique images (Figure 1F). This procedure and the full composition of the data set are described in full detail in a previous study by Oinonen et al.^25^

One example item from the generated data set is shown in Figure 1D,E. All items in the data set include a stack of 10 constant height STM and AFM images taken at 0.1 Å separation over a range of tip-sample distances starting from a close distance scan where the molecular skeleton is revealed to a distance where only the electronic structure at the Fermi level is seen. A stack of images is created as opposed to just one slice because the real tip-sample distance in the experiment is not known, and we want to avoid overfitting to an exact scanning height by letting the model see images taken at a range of different scanning heights. This is achieved by randomly selecting one two-dimensional (2D) slice from the 3D stack of images during training. Consequently, our model does not require a full stack of images at prediction time, making structure discovery easily accessible using only one image. The data set also includes three image descriptors for each molecule—atomic disks, van der Waals spheres, and height map^22^ (Figure 1D)—which we have chosen as visual and 2D representations of the molecules that we can calculate analytically from the atomic coordinates. Image descriptors are a convenient tool here, as they are not only visually intuitive for people but also suitable for many machine learning model designs. In all results, we focus on the atomic disk descriptor as it provides the most discrete predictions for the atomic positions and encodes the chemical information in the size of each disk, proportional to the covalent radius of the atom. The relative height of each atom is represented as the brightness of the disk (background is zero), and atoms deeper than 1.2 Å relative to the top atom are not considered.

### Machine Learning

In this work, the structure discovery task is formulated as an image-to-image problem, for which we have developed and trained a machine learning model which translates the STM image into a descriptor using an Attention U-Net-type model which utilizes an encoder-decoder architecture and an attention-gating mechanism.^54^ On a conceptual level, the encoder compresses the information on the input image (STM image) into a latent space vector, which in turn is translated into a spatially larger representation (image descriptor) by the decoder. The encoder consists of 4 convolutional blocks containing two 2D convolutional layers with a LeakyReLU_0.1_^55^ activation function in between, followed by batch normalization^56^ and a second LeakyReLU activation. Each of the convolutional blocks is preceded by a downscaling block, which downsamples the image using strided convolutions to increase the receptive field. The same activation and batch normalization policies are used in the downscaling. The decoder section consists of four blocks that include an upscaling block, an attention gate, and a convolutional block identical to the encoder. Upsampling is achieved using a transposed 2D convolutional layer, and in the block, the same activation and batch normalization policies are used as in the encoder. The attention gate generates highlighted regions in the input for the model to focus on using a type of skip connection where an additional query signal comes from the upscaled feature map. The attention map is concatenated with the upsampled feature map and is used as input for the convolutional block. The output layer of the model is a 1 × 1 convolutional layer followed by a ReLU activation (see full details in Figure S1).

The ML model is implemented in PyTorch,^57^ and it is trained in a supervised setting for which the images were divided into training (180,000 images), validation (20,000), and testing (35,554) sets. The model was trained for 50 epochs, and a batch size of 30 was used. The parameters of the model were optimized using the Adam optimizer^58^ to minimize the mean squared error. During training, the simulated STM images are normalized to 0 mean and unit variance, and we also add white noise and cut-outs to the images, representing electronic noise and tip artifacts to make the model not rely on pristine simulated images but instead force it to make predictions on imperfect images. After training, inference on the model can be done using true experimental images to obtain a prediction of the structure with very little computational cost.

## Data Availability

Lauri Kurki; Niko Oinonen; Adam S. Foster. Automated Structure Discovery for Scanning Tunneling Microscopy. 2023, 2312.08854. arXiv. https://arxiv.org/abs/2312.08854 (accessed March 13, 2024).

## Data Availability Statement

The source code and training data for the machine learning models is available at https://github.com/SINGROUP/ASD-STM.

## Supporting Information Available

Details of the machine learning model, constant current prediction of benzene, predictions of 3D molecules from simulated images, predictions from simulated images of the four hydrocarbons, and chemical identification (PDF)
