# Supplementary material for: Automated Structure Discovery for Scanning Tunneling Microscopy
Source: ACS Nano. 2024 Apr 22;18(17):11130–8. doi: 10.1021/acsnano.3c12654 (PMC11064214; doi:10.1021/acsnano.3c12654)
Supplement: Supplementary file 1 — nn3c12654_si_001.pdf [file nn3c12654_si_001.pdf]

Supporting Information: Automated Structure Discovery for Scanning Tunneling Microscopy

Lauri Kurki,[†] Niko Oinonen,[†,‡] and Adam S. Foster[*,†,¶]

†*Department of Applied Physics, Aalto University, 00076 Aalto, Espoo, Finland*

‡*Nanolayers Research Computing Ltd., London N12 0HL, United Kingdom*

¶*WPI Nano Life Science Institute (WPI-NanoLSI), Kanazawa University, Kakuma-machi, Kanazawa 920-1192, Japan*

E-mail: adam.foster@aalto.fi

Machine learning model

ASD-STM uses an Attention U-Net-type model originally introduced in.[S1] Overview of the model is shown in Fig. S1. It uses the U-Net architecture with an attention mechanism instead of the standard skip connection. In the encoder, the model gradually shrinks the spatial size of the input while increasing the number of channels. The process is reversed in the decoder and in the end, the spatial size of the output is the same as in the input. The implementation of the Attention U-Net in ASD-STM uses a different number of layers and channels and also processes 2 dimensional input data whereas the original model uses 3 dimensional input. Here, the query signal is also obtained from the already upsampled feature map meaning that resampling is not needed in the attention gate as in the original model.

The input layer of the model is a 2D convolutional block (Fig. S1) with 32 channels. After the input layer, at every step of the encoder there is a downscaling block and a 2D convolutional block with increasing filter dimension and decreasing spatial size. In the decoder, the output of the ConvBlock is upscaled and used as the query in the attention mechanism, where the other input comes from the skip connection. The output of the attention mechanism is concatenated with the upscaled tensor and used as input for a ConvBlock. This process is repeated four times gradually decreasing the filter dimension and increasing the spatial size of the feature map, until the dimension in $64@128 \times 128$ ($F@W \times H$, assuming 128×128 input). Finally, there is a 1×1 convolutional layer and a ReLU activation (Eq. 1) which reduce the filter dimension to 1 and translate the output values to $x \geq 0$ to match the values in the descriptor.

A ConvBlock consists of two 3×3 2D convolutional layers, first of which increases (encoder) or decreases (decoder) the number of filters. In between, there is a $\text{LeakyReLU}_{0.1}$ (Eq. 2) activation and finally a 2D batch normalization layer. A downscaling block is otherwise identical but the first convolutional layer is replaced by a 4×4 2D convolutional layer with 2-stride to reduce the spatial size of the feature map. This operation is used instead of maximum pooling that was used in the original model. Similarly, in an upscaling block the first layer is replaced with a 4×4 2D transposed convolutional layer with 2-stride to expand the feature map spatially. The final piece in the model is the attention mechanism which receives two inputs – one from the skip connection x and one from the query q. A 1×1 convolution and a batch normalization are applied to both to make the tensors one dimensional in the filter dimension, and the tensors are combined in a summing operation activated with a ReLU function to form the attention weighting vector α. Subsequently, there is a second 1×1 convolution, a batch normalization and a sigmoid activation (Eq. 3) to translate the vector into range $\alpha \in [0, 1]$. Finally, the skip connection x is multiplied with α to highlight salient regions in the feature map. The total number of parameters in the model is 51,588,013.

The activation functions used in this model are defined as follows:

$$\mathrm{ReLU}(x) = \max\left(0, x\right) \tag{1}$$

$$\mathrm{LeakyReLU}(x)_\alpha = \max\left(\alpha x, x\right), \quad \alpha \leq 1 \tag{2}$$

$$\sigma(x) = \frac{1}{1 + e^{-x}} \tag{3}$$

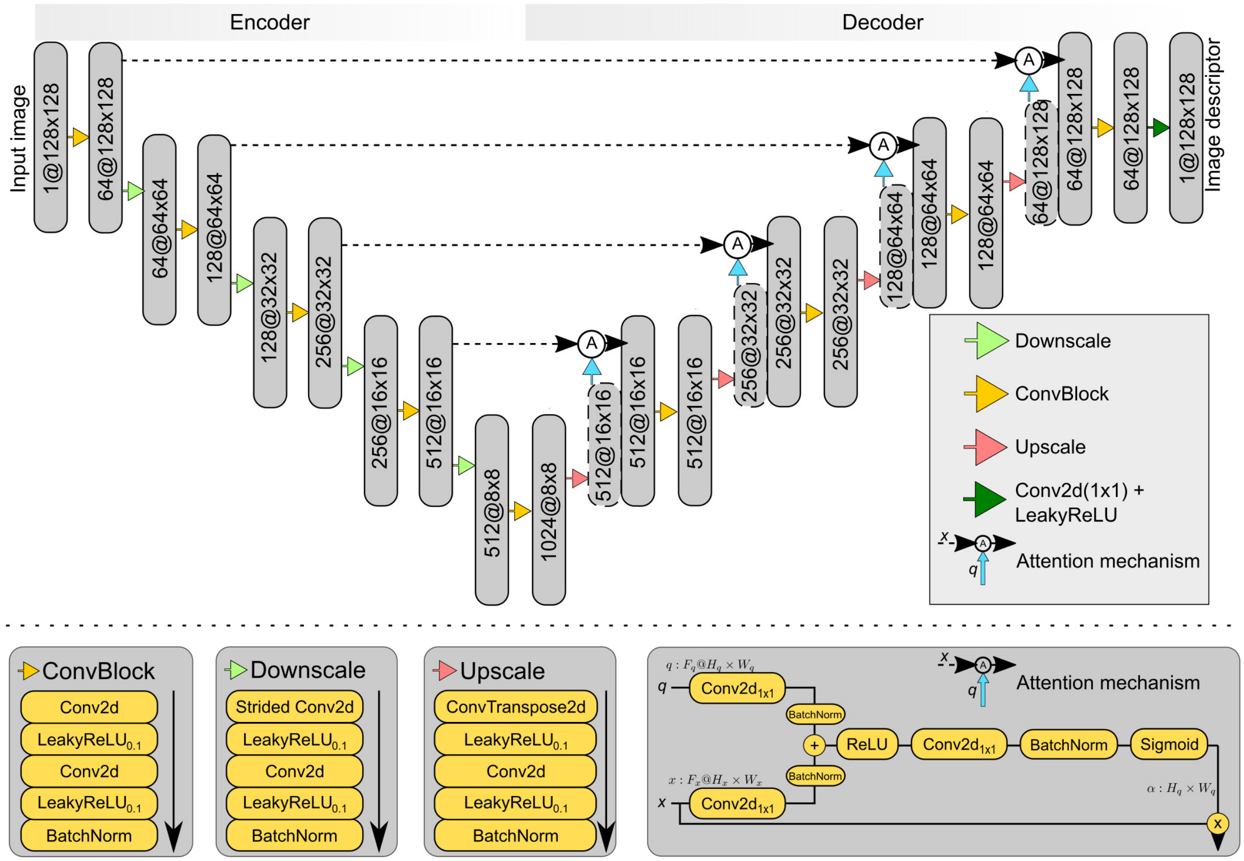

Figure S1: Diagram of the machine learning model used in ASD-STM. Different operations are denoted by colored arrows and all procedures are visualized in their respective boxes. The numbers in each block represent the number of channels and the spatial size of the feature map at every step of the model (F@W×H). Input and output are 128 × 128 images with one channel.

Three dimensional simulated examples

In addition to the examples in Figure 2, we show here eight more predictions from simulated images. These images are from the testing set meaning that the model has not seen any of these during training. These molecules were chosen to illustrate the accuracy on three-dimensional molecules and on planar molecules that are tilted with respect to the scanning plane.

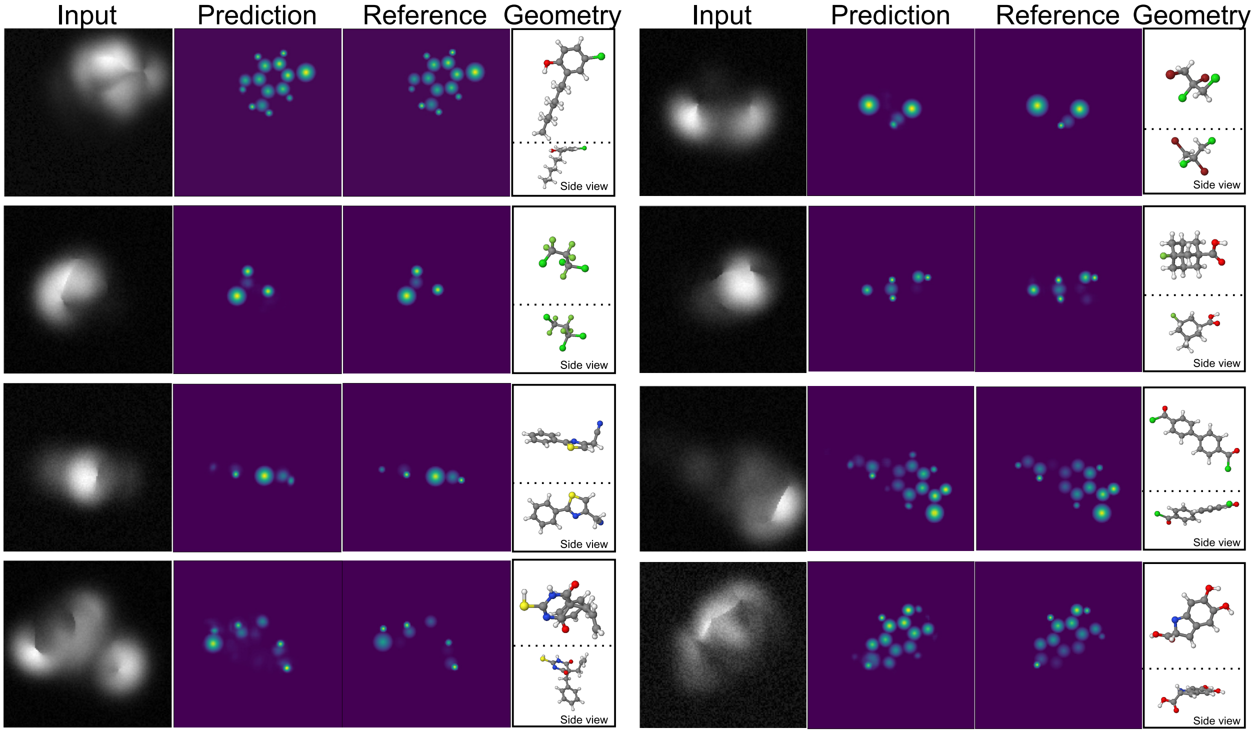

Figure S2: Eight structure predictions from simulated images of three-dimensional molecules. Each example contains the input STM image, structure prediction, reference descriptor and the geometry.

Constant current images

While PPSTM calculates exclusively constant height (CH) images, constant current (CC) images can be acquired by calculating a stack of CH images with multiple tip heights and calculating an isosurface of constant current. PPSTM doesn't provide quantitatively correct

tunneling current so simulated CC images should be fitted to experimental images to ensure accuracy. Also, choosing a set point for current is not directly comparable to experiment.

ASD-STM has also been trained on CH images and we have only validated its accuracy thoroughly on these. However, for some systems where the CC and constant CH are similar, or where the characteristic sharp lines with a CO tip appear clearly, it can be applied to CC images as well. To demonstrate this, we calculated the CC image of a benzene molecule and predicted its structure. The structure is predicted accurately but the atomic positions are not as well defined as for CH predictions. Expanding the model to include CC images would enable better predictions on three dimensional molecules and including CC images to the training data as well as full validation of ASD-STM on CC images will be looked into.

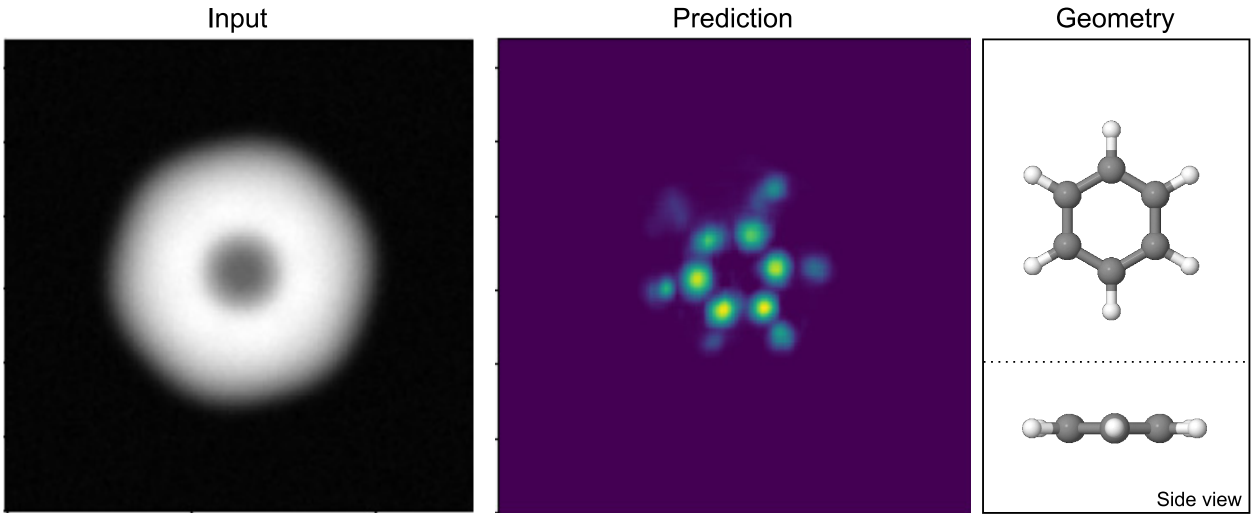

Figure S3: Simulated constant current STM image, corresponding structure prediction and the geometry.

Simulated comparison for hydrocarbons

The structure predictions of the hydrocarbons in Fig. 3 were generally accurate but there were some inaccuracies, particularly misidentified five and six-rings. While pinpointing the cause of the errors is difficult, we performed further analysis by calculating simulated STM images of the same molecules and compared the images and predictions with the experimental

counterparts. The planar structures were obtained by relaxing the molecule in isolation while constraining the z-direction.

The simulated images, predictions and relaxed structures are shown in Fig. S4. Across all molecules, the carbon backbones including all five and six-rings are predicted correctly. Comparing the STM images to the experiments, the overall features are similar but the images appear less noisy and there is more halo around the molecules. Also, the shapes of the rings are more well-defined in the simulations allowing easier identification of the ring structure. This analysis emphasizes that both the quality of training data and the specific data augmentation methods are vital in making experimental predictions as accurate as possible, and while the augmentations used here are effective in many cases, there is clearly room for advanced techniques to be developed in this area.

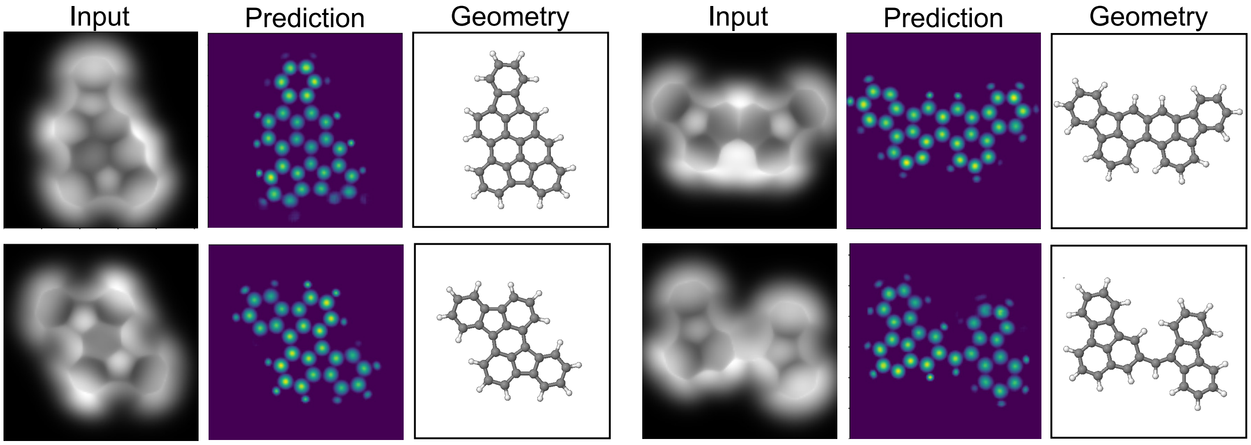

Figure S4: Structure predictions of the four hydrocarbons from simulated images for comparison. Each example contains the input STM image, structure prediction and the relaxed geometry.

Chemical identification

To assist in the chemical identification, we used the *type map* descriptor.[S2] It represents the atoms by spheres with van der Waals radii and separates the different chemical species by class into different channels (Green: H, Red: {N, O, F}, Blue: {C, Si, P, S, Cl, Br}). The

machine learning model is modified to output three channels in the last layer.

We predicted the chemical composition of the TOAT molecule from an experimental image[S3] with particular focus in distinguishing the center heteroatom (Fig. S5). Our model classified the carbon backbone and the nitrogen correctly, losing some accuracy in the perimeter atoms. We also show three type map predictions from simulated images generally achieving very good performance, including distinguishing heteroatoms within carbon rings. Different atomic species within a class, such as carbon and bromine, are identified by the size of the van der Waals sphere. To maximize the accuracy of both physical structure discovery and chemical identification, both *atomic disks* and *type map* descriptors can be utilized.

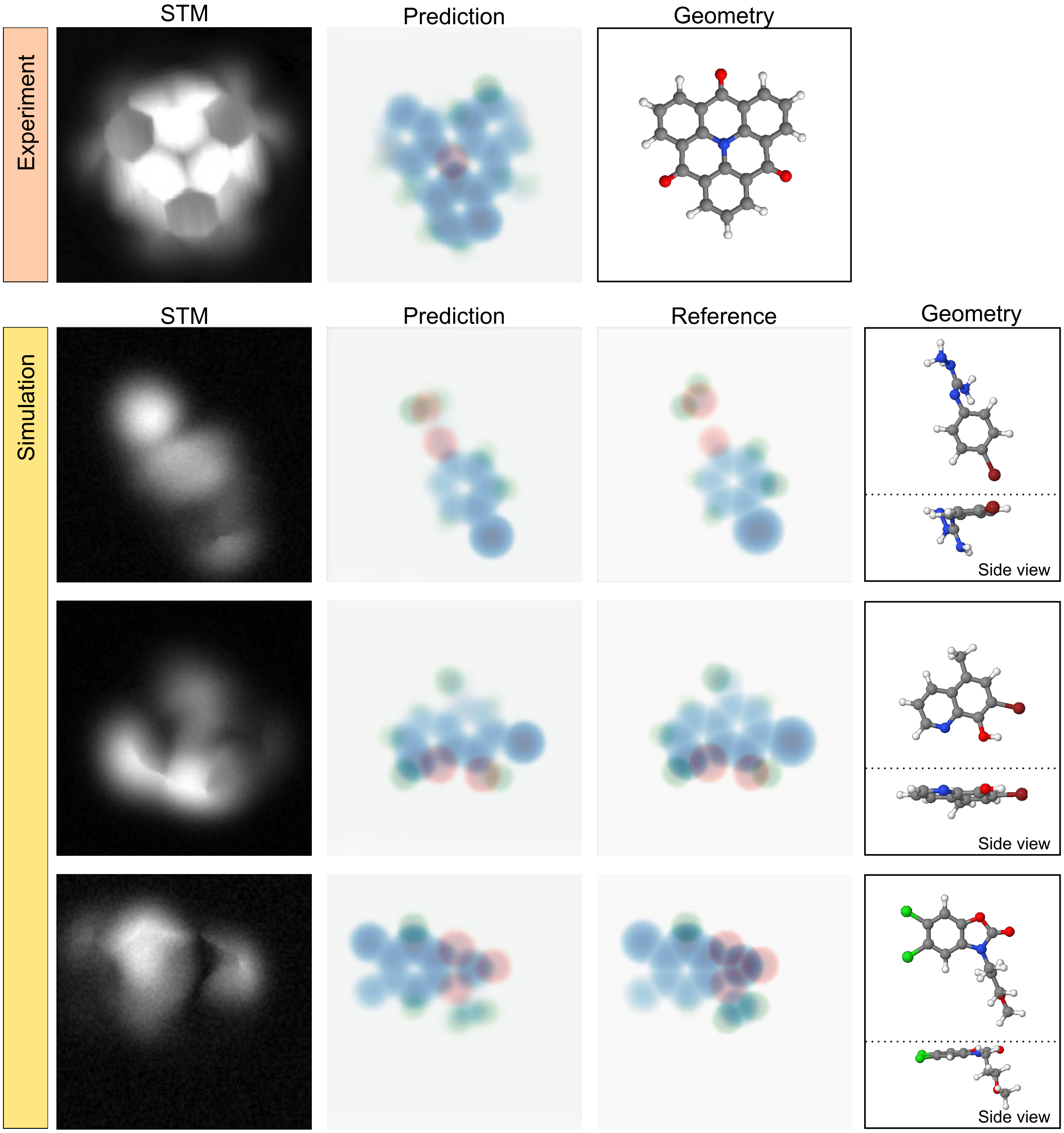

Figure S5: *Type map* predictions. Different colors correspond to different classes of atomic species. First row: prediction from an experimental image of a TOAT molecule. STM image reprinted with permission ©2016 ACS.[S3] Second row onwards: predictions from simulated images. For simulated images, the third column shows the reference descriptor.

References

(S1) Oktay, O.; Schlemper, J.; Folgoc, L. L.; Lee, M.; Heinrich, M.; Misawa, K.; Mori, K.; McDonagh, S.; Hammerla, N. Y.; Kainz, B.; Glocker, B.; Rueckert, D. Attention U-Net: Learning Where to Look for the Pancreas. 2018; 1804.03999. arXiv. https://arxiv.org/abs/1804.03999 (accessed March 13, 2024).

(S2) Alldritt, B.; Hapala, P.; Oinonen, N.; Urtev, F.; Krejci, O.; Canova, F. F.; Kannala, J.; Schulz, F.; Liljeroth, P.; Foster, A. S. Automated structure discovery in atomic force microscopy. *Sci. Adv.* **2020**, *6*, eaay6913.

(S3) Heijden, N. J. V. D.; Hapala, P.; Rombouts, J. A.; Lit, J. V. D.; Smith, D.; Mutombo, P.; Švec, M.; Jelinek, P.; Swart, I. Characteristic Contrast in Δfmin Maps of Organic Molecules Using Atomic Force Microscopy. *ACS Nano* **2016**, *10*, 8517–8525.
